# RETRACTION: Antidiabetic Activity of *Vinca rosea* Extracts in Alloxan‐Induced Diabetic Rats

**DOI:** 10.1155/ije/9763032

**Published:** 2026-06-23

**Authors:** International Journal of Endocrinology

RETRACTION: M. F. Ahmed, S. M. Kazim, S. S. Ghori, et al., “Antidiabetic Activity of *Vinca rosea* Extracts in Alloxan‐Induced Diabetic Rats,” *International Journal of Endocrinology*, 2010, 841090, https://doi.org/10.1155/2010/841090.

The above article, published online on 22 June 2010 in Wiley Online Library (https://wileyonlinelibrary.com), has been retracted by agreement between the journal Editor‐in‐Chief, Zhongjian Xie, and John Wiley & Sons Ltd. The retraction has been agreed due to concerns raised with Figure 1d, as reported on PubPeer [1]. Specifically, Figure 1d shares unexpected, repeated regions with the Insulin (x10)/lrs2‐/‐ panel in Figure 2a of [2].

The authors were contacted about the concerns and responded stating that, because the study was conducted over 17 years ago, they no longer had either the original raw data or related electronic files. After assessment of the author’s response, the editors feel that the results and conclusions can no longer be considered reliable, and the article is therefore retracted.

The authors did not respond to the retraction.
